# Re-audit of use of seclusion in a tier 4 adolescent psychiatric intensive care unit

**DOI:** 10.1192/bjo.2021.319

**Published:** 2021-06-18

**Authors:** Yuki Takao, Francesca Davis, Ivan Saeger, Sophia Ulhaq, Rafik Refaat

**Affiliations:** East London Foundation Trust

## Abstract

**Aims:**

To re-audit seclusion practices within a Tier 4 Adolescent PICU provision in London, originally audited in 2018. To ensure restrictive practices are only used in exceptional circumstances for short term risk management. To evaluate whether practice has improved following introduction of incidence reduction strategies and identify any further areas of development.

**Background:**

This Tier 4 Adolescent PICU provides treatment of up to 16 high risk and unwell adolescents with severe and enduring mental health illnesses. Seclusion should be a short term risk management strategy with subsequent review of the care plan and treatment. It should be used for the shortest time possible. Following the audit in 2018, three strategies were implemented to reduce restrictive practice: (1) daily nursing safety huddles, (2) weekly Incidence Reduction meetings, and (3) ongoing QI project on restrictive practice.

**Method:**

Data were collected from all patients requiring seclusion between January and December 2019 (n = 18), which included 46 incidents. Data were collected from RiO computer records, extracting details of patient demographics, reasons and context of seclusion, risk reduction steps prior, length of seclusion, monitoring, and modifications to care plans.

**Result:**

Average length of stay in seclusion was 20h, reduced from 30h previously. Over half of patients requiring seclusion had symptoms of psychosis, consistent with the original audit. Majority of incidents involved assault to staff (80.4%) as indication for seclusion, compared to 50% previously. In 58.7% of cases, verbal de-escalation was followed by further risk reduction with oral medication. Overall, rapid tranquillisation was required in 45.7% of incidents. Restraint was used in 84.8% of incidents, always in combination with at least one other management strategy.

Just under half of seclusions were monitored and documented in line with Trust guidelines, however, there was significant improvement in documentation of consultant reviews within 24h from under 70% to over 90%. Care plan modification rates improved from 63% to over 95%.

**Conclusion:**

Majority of seclusion incidents were due to violent acts by young people presenting with psychotic features/disorder. This reflects the complex nature of psychosis and the substantial need for research to reduce restrictive practice in such cases.

Ongoing review of data relating to seclusion will continue to inform and improve practice. This re-audit demonstrates improvement in various areas after implementation of strategies to reduce restrictive practice – importantly, average time in seclusion, documentation of 24 hour consultant reviews and focus on non-pharmacological risk reduction approaches in care plan modifications.

